# A Comparative Analysis of E-cigarette Users and State-Specific Prevalence Change in the United States Between 2017 and 2018

**DOI:** 10.7759/cureus.12079

**Published:** 2020-12-14

**Authors:** Tarang Parekh, Rupak Desai

**Affiliations:** 1 Epidemiology and Public Health, George Mason University, Fairfax, USA; 2 Cardiology, Atlanta Veterans Affairs Medical Center, Decatur, USA

**Keywords:** e-cigarette, vaping, smoking, marijuana, state prevalence, e-cigarette laws, demographics, electronic cigarette

## Abstract

Introduction

Despite states’ regulatory efforts, e-cigarettes are gaining popularity, which poses a public health concern. The study objective is to compare demographic and state prevalence changes in e-cigarette use from 2017 to 2018.

Methods

A retrospective analysis was conducted using publicly available data from the Behavioral Risk Factor Surveillance System survey (2017-2018). The prevalence of current e-cigarette use was analyzed with direct age-adjustment based on the 2010 United States Census population.

Results

The overall use of e-cigarettes increased from 4.3% in 2017 to 5.4% in 2018. Although most demographics reported increased prevalence from 2017 to 2018, the most significant change was observed in younger adults (18-24), males, Hispanics, college graduates, non-smokers, marijuana non-users, and heavy alcoholics. Oklahoma (9.8%), Hawaii (7.8%), Arkansas (7.7%), and Colorado (7.3%) greater prevalence in 2018. Significant inclining prevalence was observed in Alaska, Connecticut, and Massachusetts, while Illinois reported a sharp decline. California, the District of Columbia, and Puerto Rico consistently reported the lowest prevalence. Idaho, Maine, Michigan, North Dakota, and Oregon are transitioning to a higher prevalence of e-cigarette use from 2017 to 2018.

Conclusion

The rising prevalence of e-cigarettes across demographics warrants a holistic approach to behavioural change interventions, health awareness and education, and regulatory efforts.

## Introduction

The most frequently published data on substance use in recent times remain pertinent to electronic cigarettes (vaping) and cannabis (marijuana) use in addition to tobacco smoking and alcohol abuse among young [1,2]. The paucity of evidence proposing a clear risk and benefits of mainly using e-cigarettes merits for contemporary data to screen population remaining at higher risk of polysubstance use with potentially greater health risk. We sought to assess the age-adjusted prevalence of current e-cigarette use between 2017 and 2018 in a diverse subset of the population. Concomitantly we report comparative state-specific prevalence in 2017 and 2018 with state-wise effective dates on laws related to e-cigarettes.

## Materials and methods

A retrospective analysis was conducted using the publicly available data from the Behavioral Risk Factor Surveillance System (BRFSS) survey (2017-2018). The BRFSS is the largest telephone-based annual population survey of United States adult residents conducted by the Centers for Disease Control and Prevention (CDC) on health-related risk behaviours, chronic health conditions, and preventive services utilization [3]. The prevalence of current e-cigarette use was analyzed with direct age-adjustment based on the 2010 US Census population. Data were collected from main and all available optional modules with information on e-cigarette use. Current e-cigarette use was assessed using the following two BRFSS questions: (1)“Have you ever used an e-cigarette or other electronic vaping products, even just one time, in your entire life?” and (2)“Do you now use e-cigarettes or other vaping products every day, some days, or not at all?” Respondents who answered yes to the first question were classified as current users if they used e-cigarettes daily or some days in the second question. The demographics and behavioural characteristics of e-cigarette users were selected based on previously published studies from the same database [1,4,5]. The information on state-wise e-cigarette regulations as of July 2020 was gathered from publicly available sources [6,7].

Baseline characteristics for the current e-cigarette use were compared using row frequencies with appropriate BRFSS survey weights and developed methodology [8,9]. Analyses were conducted using Stata-16.1, and maps were created using ArcGIS-10.7.1.

## Results

A total of 31,818 participants reported using e-cigarettes from 2017 to 2018. The overall use of e-cigarette increased from 4.3% in 2017 (95%CI: 4.2%-4.5%) to 5.4% in 2018 (95%CI: 5.2%-5.6%). The prevalence increased by 46.9% (Annual Percentage Change, APC) among younger adults aged 18-24 years from 9.6% in 2017 to 14.1% in 2018. Hispanics (APC 68.9%) and college graduates (APC 57.1%) also reported a greater increase in e-cigarette use. By smoking status, never (APC 46.2%) and former (APC 40.2%) smokers reported greater incline compared to current smokers (APC 12.7%). E-cigarette use increased by 45.8% among marijuana non-users from 2.4% in 2017 to 3.5% in 2018, higher than the observed increase in marijuana users (APC 19.4%). Of reported demographics, only underweight individuals (APC -16.2%) and pregnant women (APC -21.7%) showed a lower prevalence of e-cigarette use in 2018 than in 2017 (Table 1).

**Table 1:** Age-adjusted prevalence and prevalence percentage change in current e-cigarette use among adults aged 18-64 years, Behavioral Risk Factor Surveillance System, 2017-2018 ^a ^Unadjusted weighted prevalence reported. ^b^ Data not available for the entire BRFSS data years. Reported prevalence is calculated from the secondary data analyses using respected sub-population characteristics. ^c ^Heavy alcohol use reported for males having five or more drinks on one occasion and females having four or more drinks on one occasion at least once in a month. ^d^ Smokeless tobacco use is calculated if an individual reported current use of chewing tobacco, snuff, or snus every day or some days. ^e^ Physically active participants were identified if individuals reported any physical activity or exercises (ex. running, calisthenics, golf, gardening, or walking) other than their regular job last month.

Characteristics	Age-adjusted prevalence of current e-cigarette use, % (95% CI)		Annual Percentage Change (APC), %
	2017 (n= 570,589)	2018 (n= 360,096)	2017-2018
Overall	4.3 (4.2- 4.5)	5.4 (5.2 - 5.6)	25.6
Age Group ^a^			
18-24 year	9.6 (8.9 - 10.3)	14.1 (13.1 - 15.1)	46.9
25-34 year	6.6 (6.9 - 7.1)	7.7 (7.1 - 8.4)	16.7
25- 44 year	4.4 (4.1 - 4.8)	5.3 (4.8 - 5.9)	20.5
45- 54 year	3.1 (2.8 - 3.4)	3.6 (3.2 - 3.9)	16.1
55- 64 year	2.5 (2.2 - 2.7)	2.7 (2.3 - 3.0)	8.0
65 or above year	0.9 (0.8 - 1.0)	1.0 (0.8 - 1.1)	11.1
Sex			
Male	5.4 (5.1 - 5.6)	6.8 (6.4 - 7.1)	25.9
Female	3.2 (3.1 - 3.4)	4.0 (3.8 - 4.3)	25.0
Race			
White, NH	5.5 (5.3 - 5.7)	6.7 (6.4 - 7.0)	21.8
Black, NH	3.0 (2.6 - 3.4)	3.3 (2.9 - 3.8)	10.0
Other, NH	3.6 (3.2 - 4.1)	4.9 (4.2 - 5.7)	36.1
Hispanic	2.2 (1.9 - 2.5)	3.4 (2.9 - 3.9)	54.6
Gender Orientation ^b^			
Cisgender	4.1 (3.9 - 4.4)	5.8 (5.5 - 6.1)	41.5
Transgender	6.1 (3.4 - 8.9)	10.3 (6.9- 13.8)	68.9
Sexual Orientation ^b^			
Straight	4.1 (3.8 - 4.3)	5.7 (5.4 - 6.0)	39.0
Lesbian/Gay	6.3 (4.6 - 8.0)	8.6 (6.7 - 10.5)	36.5
Bisexual	6.9 (5.3 - 8.5)	8.9 (7.3 - 10.5)	29.0
Other	5.7 (2.3 - 9.2)	7.1 (4.8 - 9.3)	24.6
Education			
Less than High School	4.5 (4.0 - 4.9)	5.2 (4.6 - 5.9)	15.6
High School Graduate	5.4 (5.1- 5.7)	6.6 (6.1 - 7.0)	22.2
Some College	5.1 (4.8 - 5.4)	6.4 (5.9 - 6.8)	25.5
College Graduate	2.1 (1.9 - 2.4)	3.3 (3.0 - 3.6)	57.1
Marital Status			
Married	3.1 (2.9 - 3.4)	3.7 (3.3 - 4.2)	19.4
Unmarried	4.9 (4.6 - 5.3)	6.4 (6.0 - 6.9)	30.6
Divorced/Widow/Separated	6.5 (5.6 - 7.4)	8.3 (6.9 - 9.7)	27.7
Employment Status			
Unemployed	6.0 (5.3 - 6.7)	6.7 (5.5 - 7.9)	11.7
Employed	4.3 (4.1 - 4.5)	5.5 (5.2 - 5.8)	27.9
Retired	3.2 (1.1 - 5.4)	3.2 (1.6 - 4.8)	0.0
Student/Home Maker	3.0 (2.7 - 3.4)	3.7 (3.2 - 4.2)	23.3
Unable to Work	6.9 (6.0 - 7.8)	9.4 (7.5 - 11.2)	36.2
Cigarette Smoking			
Never Smoker	1.3 (1.1 - 1.4)	1.9 (1.7 - 2.0)	46.2
Former Smoker	8.7 (8.0 - 9.3)	12.2 (11.3 - 13.0)	40.2
Current Smoker	13.4 (12.7 - 14.1)	15.1 (14.2 - 16.0)	12.7
Heavy Alcohol Use ^c^			
No	4.1 (3.9 - 4.2)	5.0 (4.7 - 5.2)	21.9
Yes	7.6 (6.8 - 8.4)	10.5 (9.5 - 11.5)	38.2
Smokeless Tobacco Use ^d^			
Never	4.1 (3.9 - 4.3)	5.2 (4.9 - 5.4)	26.8
Sometimes	11.5 (9.6 - 13.4)	14.7 (12.4 - 17.1)	27.8
Regular	7.6 (5.9 - 9.3)	8.1 (6.2 - 10.0)	6.6
Current Marijuana Use ^b^			
No	2.4 (2.1 - 2.8)	3.5 (3.1 - 3.9)	45.8
Yes	9.3 (7.3 - 11.2)	11.1 (9.6 - 12.6)	19.4
Physically Active ^e^			
No	4.8 (4.4 - 5.1)	5.5 (5.0 - 6.0)	14.6
Yes	4.1 (4.0 - 4.3)	5.3 (5.1 - 5.5)	29.3
Body Mass Index (BMI)			
Underweight	7.4 (5.8 - 9.0)	6.2 (4.8 - 7.5)	-16.2
Normal	4.4 (4.1 - 4.6)	5.6 (5.2 - 5.9)	27.3
Overweight	4.5 (4.2 - 4.8)	5.3 (4.9 - 5.7)	17.8
Obese	4.7 (4.3 - 5.0)	5.6 (5.1 - 6.1)	19.2
Pregnancy Status			
Non-pregnant	3.0 (2.6 - 3.5)	4.3 (3.0 - 5.6)	43.3
Pregnant	2.3 (0.1 - 4.7)	1.8 (1.0 - 2.6)	-21.7
Veteran			
No	4.2 (4.0 - 4.4)	5.2 (5.0 - 5.4)	23.8
Yes	6.0 (5.4 - 6.7)	8.4 (7.0 - 9.7)	40.0

Table 2 showed the state-specific prevalence of e-cigarette use and effective dates for relevant e-cigarette laws. While the majority of states have passed e-cigarette related laws very recently that limits the ability to assess the effectiveness of such laws, the table provides critical information for future study references.

**Table 2:** Annual Prevalence change in e-cigarette use by states, and effective dates for e-cigarette related laws ^a^ State laws effective data for any excise tax levied on e-cigarettes. ^b^ Effective date from when an individual is required to obtain a license or permit before conducting the business of selling e-cigarettes over the counter. ^c^ Effective date from when state laws prohibit smoking in indoor areas of private worksites, restaurants, and bars. ^d^ Effective date for required minimum 21 years of age by a state that an individual must reach before vendors can legally sell e-cigarettes to an individual. * Date when state law was enacted. The effective date is not available.

State	Prevalence, % (2017)	Prevalence, % (2018)	Annual Percentage Change, %	Laws on Taxing E-cigs ^a^	Laws on E-cig Retail Licensure ^b^	Laws on Prohibiting indoor e-smoking ^c^	Laws on sales restriction to age <21 ^d^
Alabama	5.2	6.4	18.8		8/1/2019		
Alaska	3.2	6.2	48.4		1/1/2019		
Arizona	5.5	3.1	-77.4				
Arkansas	5.8	7.7	24.7		5/1/2015		12/31/2021
California	3.1	3.9	20.5	4/1/2017	1/1/2017	6/9/2016	6/9/2016
Colorado	5.4	7.3	26.0			7/1/2019	
Connecticut	3.5	6.1	42.6	10/1/2019	3/1/2016		10/1/2019
Delaware	5.2	5.2	0.0	1/1/2018		10/5/2015	7/16/2019
District of Columbia	2.2	2.0	-10.0	10/1/2015	10/22/2015	11/18/2016	11/29/2016
Florida	4.8	7.0	31.4				
Georgia	4.4	5.6	21.4				
Hawaii	5.0	7.8	35.9		7/1/2018	1/1/2016	1/1/2016
Idaho	4.7	6.5	27.7		7/1/2020		
Illinois	4.9	2.4	-104.2	7/1/2019			7/1/2019
Indiana	6.1	7.1	14.1		7/1/2015		
Iowa	4.2	5.7	26.3		7/1/2014		
Kansas	4.6	5.8	20.7	7/1/2017	7/1/2012		
Kentucky	6.1	6.8	10.3				
Louisiana	4.5	6.1	26.2	7/1/2015	5/28/2014		
Maine	4.6	6.2	25.8	1/2/2020	11/1/2017		7/1/2021
Maryland	3.4	5.0	32.0		10/1/2017		10/1/2019
Massachusetts	3.5	5.9	40.7	6/1/2020	6/1/2020	7/27/2018	12/31/2021
Michigan	4.9	6.4	23.4				
Minnesota	3.8	5.4	29.6	8/1/2010	8/1/2014		
Mississippi	5.1	5.9	13.6				
Missouri	5.3	6.2	14.5		10/10/2014		
Montana	4.4	5.2	15.4		1/1/2016		
Nebraska	3.8	5.8	34.5		1/1/2020		
Nevada	5.6	6.0	6.7	1/1/2020	1/1/2020		
New Hampshire	5.0	5.4	7.4	1/1/2020	7/1/2019		
New Jersey	4.8	N/A	N/A	9/29/2018	11/1/2019	7/11/2010	11/1/2017
New Mexico	5.1	4.5	-13.3	7/1/2019	1/1/2021	6/14/2019	
New York	4.0	5.6	28.6	12/1/2019	12/1/2019	11/22/2017	11/13/2019
North Carolina	5.0	5.7	12.3	6/1/2015			
North Dakota	4.0	6.0	33.3			12/6/2012	
Ohio	5.7	5.9	3.4	10/1/2019	7/18/2019		10/17/2022
Oklahoma	6.9	9.8	29.6				
Oregon	4.7	6.0	21.7			1/1/2016	1/1/2018
Pennsylvania	5.1	N/A	N/A	7/13/2016	7/13/2016		7/1/2020
Rhode Island	5.1	5.8	12.1		1/1/2015	7/1/2018	
South Carolina	4.3	3.2	-34.4				
South Dakota	4.3	4.8	10.4			7/1/2019	
Tennessee	6.2	6.5	4.6				
Texas	4.6	5.6	17.9		10/1/2015		8/31/2022
Utah	4.7	5.3	11.3	7/1/2020	7/1/2015	5/8/2012	7/1/2021
Vermont	3.4	4.1	17.1	7/1/2029	7/1/2013	7/1/2016	9/1/2019
Virginia	5.1	5.3	3.8				7/1/2019
Washington	4.4	N/A	N/A	10/1/2019	6/28/2016		1/1/2020
West Virginia	6.3	N/A	N/A	7/1/2016			
Wisconsin	4.5	5.1	11.8	10/1/2019			
Wyoming	6.0	7.0	14.3	7/1/2020	7/1/2020		7/1/2020
Guam	5.8	5.0	-16.0				1/1/2018
Puerto Rico	1.2	1.5	20.0	4/29/2017		4/11/2011	
Virgin Islands	N/A	N/A	N/A	10/15/2014*			

**Figure 1:**
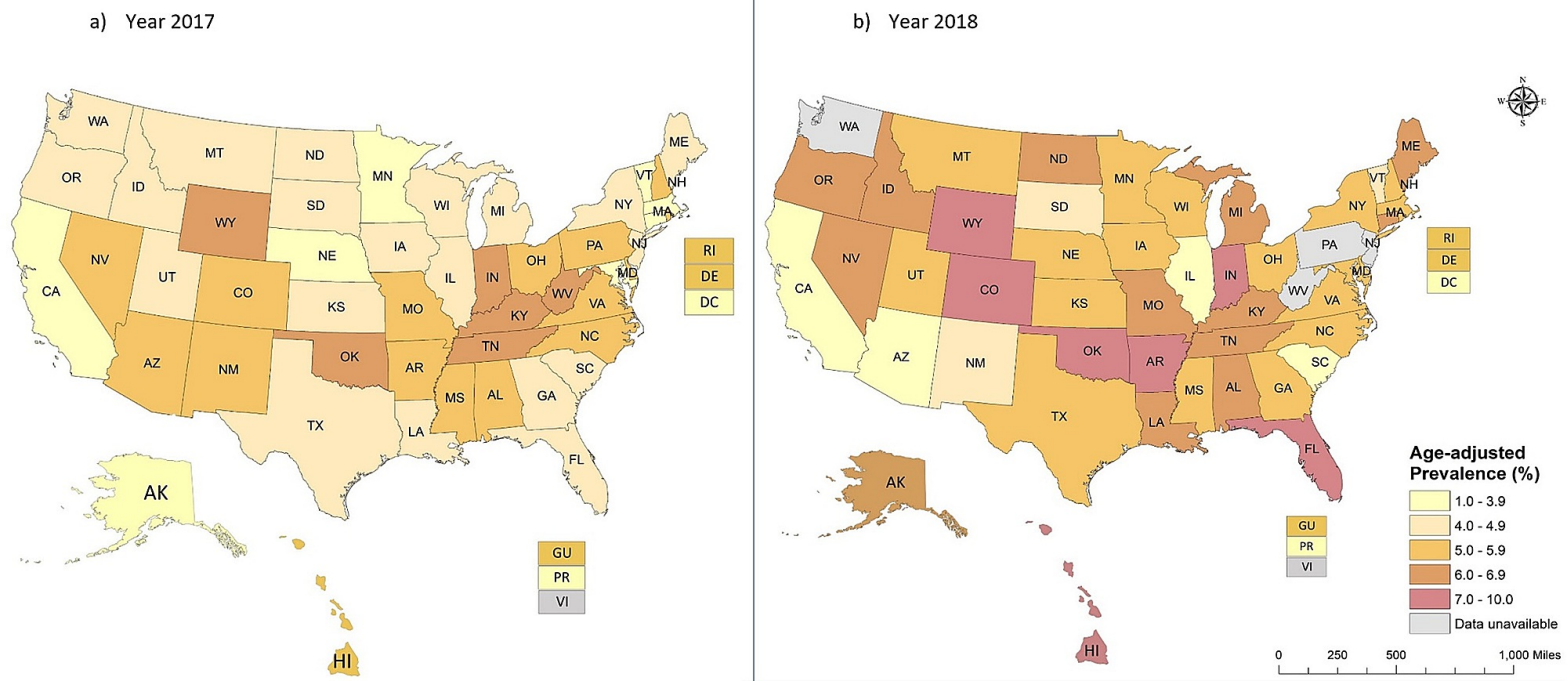
State-specific e-cigarette prevalence A: Year 2017 B: Year 2018

Overall, the majority of states reported increased prevalence in 2018 than in 2017 (Figure 1). In 2018, the highest prevalence was observed in Oklahoma (9.8%), Hawaii (7.8%), Arkansas (7.7%), Colorado (7.3%), Indiana (7.1%), Wyoming (7.0%), and Florida (7.0%). The lowest prevalence (<4%) was reported in Puerto Rico, District of Columbia, Illinois, Arizona, and California. Of states, Alaska (APC 48.4%), Connecticut (APC 42.6%), and Massachusetts (APC 40.7%) reported significant inclining, while Illinois (APC -104.2%) and Arizona (APC - 77.4%) reported a major declining prevalence of e-cigarettes from 2017 to 2018. Idaho, Maine, Michigan, North Dakota, and Oregon states are transitioning to a higher prevalence of e-cigarette use from 2017 to 2018.

## Discussion

Recent reports on the prevalence of e-cigarette use showed varied rates in the United States adult population representing diverse geographic, ethnic, and socioeconomic strata [1]. Consistently, we observed Caucasians dominating the study cohort; however, all age, sex, and racial/ethnic groups reported increased e-cig use during the study period. The most pronounced increase was noted among the young (18-24 years) participants, males, and Hispanics. Education and employment status also showed an impact on the frequency of e-cigarettes. Low education among participants (less than high school, 4.5% & 5.2%) and those who were either unable to work (6.9% & 9.4%) or were unemployed (6% & 6.7%) reported a high frequency of using e-cigarettes. Expectedly, current cigarette smoking was associated with a higher frequency (15.1%) of concomitant e-cigarette smoking as compared to former cigarette smoking. This finding is corroborative of earlier reports suggesting concerning behavioural patterns in youth indicating polysubstance use. While the prevalence of e-cigarette use during the last two years was also evident in heavy alcohol users, marijuana users, current smokers, the greatest inclining was observed among marijuana non-users and non-smokers. This rising frequency of e-cigarettes use is concerning as numerous reports starting suggested potential severe adverse healthcare effects of using e-cigarettes, including cardio-cerebrovascular and lung-injury [4,10,11].

Furthermore, we found interesting differences in terms of the state-wise prevalence of e-cigarette use during the study period. Looking at higher incremental use of e-cigarettes in states including Arkansas, Colorado, Florida, Indiana, Oklahoma, and Wyoming, call for attention from policymakers especially any conclusive evidence showing beneficial effects of e-cigarettes over combustible cigarette smoking over a long period whereas numerous reports suggesting acute healthcare effects in young individuals using e-cigarettes [4,10,12]. Reassuringly, a few states including Arizona, Illinois, New Mexico, and South Carolina, showed a steep decline in the use of e-cigarettes which could be hypothesized due to improved awareness regarding the ill effects of vaping among the general public and physicians. However, it would be interesting to see if these states would be able to continue to control the vaping epidemic until clear, conclusive evidence and guidelines endorse the use of e-cigarette use and its benefits over combustible cigarette use. Interestingly, California, the District of Columbia, and Puerto Rico have shown consistently lower prevalence during 2017-18. One possible explanation could be laws taxing e-cigarettes and strict e-smoking regulations, including required retail licensure imposed before the study period [6,7,13]. In contrast, states such as Idaho, Michigan, North Dakota, and Oregon without requirements for retail licensure and excise tax showed increasing prevalence from 2017 to 2018. Future studies should look at the longitudinal effects of states’ policy efforts in regulating e-cigarette use and changing prevalence in the population. 

Although the survey design is limited in terms of self-reported information and identifying the duration, mode, and sequential use of the e-cigarette, combustible cigarette, and marijuana, it remains important to highlight the alarming rate at which polysubstance abuse has been increasing since the last few years. While it is early to conclude the relationship between e-cigarette, smoking, and marijuana, it is essential to assess ongoing trends between 2019 and 2020 especially with enormous scientific evidence highlighting the severe health impacts of both substances.

## Conclusions

The study provides essential information on a recent increase in the overall use of e-cigarette and concomitant rising prevalence of polysubstance use. The most pronounced rise in e-cigarette use between 2017 and 2018 was noted in young, male, and Hispanic survey participants. A few states like California and the District of Columbia showed an encouraging decline in e-cigarette use. In contrast, states like Arkansas, Colorado, and Florida showed a steep rise and call for attention from physicians and policymakers to spread awareness among young adults about the adverse effects of e-cigarette use. Whether state regulation efforts have any influence on e-cigarette prevalence is inconclusive, these findings open a door for debate on reevaluating states’ current smoking policies and public health efforts.
